# Supplementary figures and images for: Integrated Information Increases with Fitness in the Evolution of Animats
Source: PLoS Comput Biol. 2011 Oct 20;7(10):e1002236. doi: 10.1371/journal.pcbi.1002236 (PMC3197648; doi:10.1371/journal.pcbi.1002236)

A

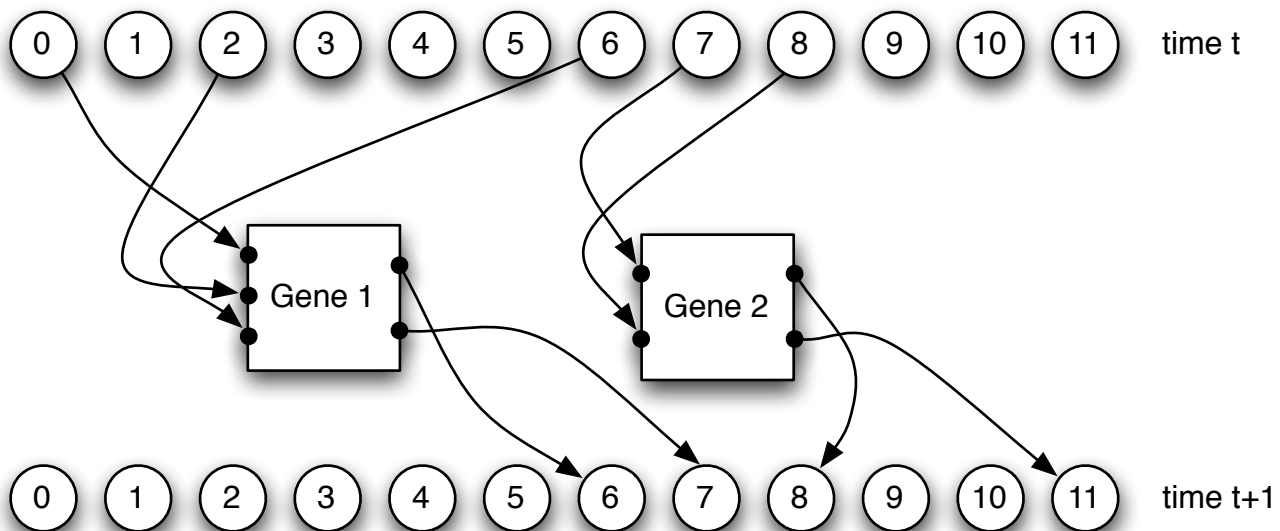

B

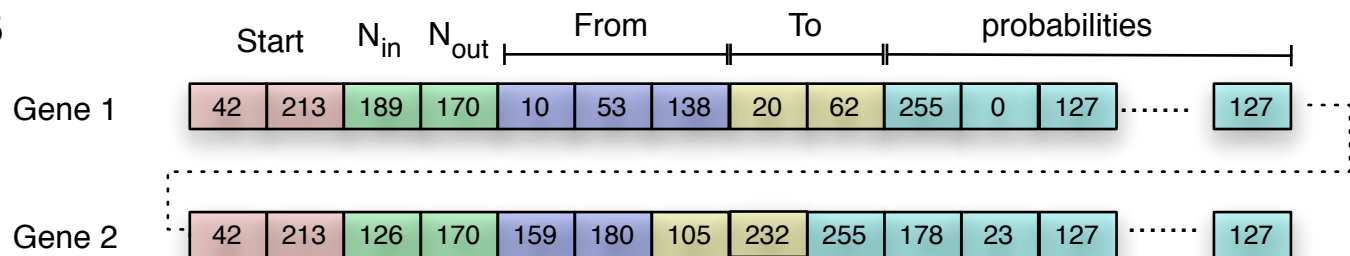

C

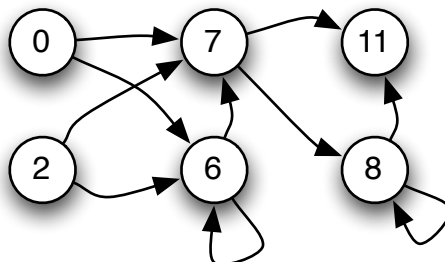

Supplement: Figure S1 — Genetic encoding of animat controllers. A: In this example, two HMGs encoded by two genes can read from and write to several of the 12 Markov variables, indexed 0–11. The top row shows the Markov variables at time that the HMGs can read from while the row below shows how the HMGs write into those variables to update their state at . B: The genome is a circular sequence of loci that carry unsigned integers and encode the input output structure of each HMG as well as the connectivity between them and the state transition tables that determine each HMG's function. Colors denote different functional sections of the gene. C: Causal influence of the Markov variables induced by the two HMGs. Presence of an arrow between variables and implies that may change the state of in a single time step. Absence of an arrow implies that the variables cannot influence each other within a single time step. (PDF) [file pcbi.1002236.s001.pdf]
