# Supplementary material for: Integrated Information Increases with Fitness in the Evolution of Animats
Source: PLoS Comput Biol. 2011 Oct 20;7(10):e1002236. doi: 10.1371/journal.pcbi.1002236 (PMC3197648; doi:10.1371/journal.pcbi.1002236)
Supplement: Text S1 — Relationship between different forms of (PDF) [file pcbi.1002236.s002.pdf]

Text S1. Relationship between different forms of I_{pred}

Our definition of I_{pred} as the shared entropy between sensor variables at time t and actuator variables at time $t+1$ ostensibly differs from that of Bialek et al. [35] and [36], as well as from measures quantifying the value of information used by Rivoire and Leibler [27]. This difference is due to the particular way in which our animats interpret sensorial signals via their action variables and the dynamics of how the animat and the environment are updated, but are otherwise directly comparable. Bialek et al. [35] define I_{pred} as the shared entropy between the states of a data stream X in the past and in the future, which for environments with the Markov property reduces to

$$I_{\text{pred}} = I(X_t : X_{t+1}) \;, \tag{S1}$$

which is the shared Shannon entropy between subsequent states of the data stream. Ay et al. [36] adapted this measure to the sensor-action loop of an autonomous robot where motor variables Y_t affect the sensed variables X_{t+1} one update later, rewriting the predictive information Eq. (S1) in terms of sensor and motor variables as

$$I_{\text{pred}} = I(Y_t : X_{t+1}) \;. \tag{S2}$$

This differs from our Eq. (1) when identifying $X_t \equiv S_t$ and $Y_t \equiv R_t$ because of different ways in which the dynamics of the systems are updated. In [36], the authors advance the time counter when the actions of the controller are applied to the environment, so that

$$x_{t+1} = F(x_t, y_t) + \xi_{t+1} \;, \tag{S3}$$

where ξ_{t+1} is a Gaussian white noise term and $F(x,y)$ is a function mapping old sensor and motor variables to their updated values. Instead, we advance the time counter when the motor variables are updated based on the sensed environment:

$$y_{t+1} = G(x_t, y_t) \tag{S4}$$

with a different update function $G(x_t, y_t)$ that is optimized by evolution and contains stochastic effects from the HMGs. With this updating scheme, the sensed values are the cause and the changed motor variables are the effect while using the update scheme of Ref. [36], the motor variables are the cause and the change in sensor values is the effect. Both versions, however, capture the predictive information. Note that when $F(x_t, y_t) = x_t$ in (S3) as in Ref. [36], expression (S2) reduces to (S1).

Rivoire and Leibler discuss simple models of populations that use environmental states x_t to optimize their growth. In case no information is inherited (or remembered), the fitness of the population is maximized where the Shannon information $I(X_t : Y_t)$ is maximal. Here, the states y_t are environmental signals that the agent perceives *and uses* in order to survive optimally in the environment. In that respect, they correspond to those environmental cues that had an effect on the behavior of the agent; thus they are best described by motor variables at time $t+1$ (sensed values that do not affect the motors could as well have been random). Thus, in the absence of inherited information or memory, the Shannon information $I(X_t : Y_t)$ is equivalent to our predictive information Eq. (1) in the main text. If memory or other information influences the actions of the agent, $I(X_t : Y_t)$ no longer maximizes the fitness. In a simple model where agents act optimally given the environment, the fitness is maximal at high $I(X_t : Y_t|X_{t-1})$, but if the strategy is non-optimal (as in the case we discuss here), no general expression can be given [27].
