# Supplementary material for: Integrated Information Increases with Fitness in the Evolution of Animats
Source: PLoS Comput Biol. 2011 Oct 20;7(10):e1002236. doi: 10.1371/journal.pcbi.1002236 (PMC3197648; doi:10.1371/journal.pcbi.1002236)
Supplement: Text S2 — Genetic encoding of network structure and function. (PDF) [file pcbi.1002236.s003.pdf]

Text S2. Genetic encoding of network structure and function

Hidden Markov Gates (HMGs) are encoded by genes that specify where their inputs come from (what Markov variable is read at time t) and where the gate writes to, thus updating that Markov variable at time $t+1$. The 12 (binary) variables that we use can be depicted at a point in time t as in Fig. S1A (top). Because HMGs can read or write from these variables, write events from multiple HMGs have to be resolved in order for the variable to take on a unique state at each t. This is achieved by combining the inputs to a variable via an OR operation. We allow at most 3 simultaneous write attempts into each variable. An HMG that attempts to write into a variable that already has 3 connections to it has its connection attempt redirected to the nearest variable that has open slots remaining (such redirects are rare). Before the Markov variables are updated, they are all cleared (internal variables as well as sensors and actuators), so that no information can be stored by simply not writing into a variable before it is read. Rather, memory has to emerge using the computational structure of the network. In this sense, the 12 Markov variables are completely passive conduits for information processing.

An HMG is encoded by a gene that is identified by a "Start" sequence given by the specific alleles '42' followed by '213' (see Fig. S1B). The start signal is chosen so as not to interfere with common alleles appearing in the genome, such as the numbers '0', or '255'. The exact "start codon" (42,213) is therefore rare, occurring by chance only once every 2^{16} pairs. The next two loci encode the number of inputs $N_{\rm in}$ and $N_{\rm out}$ of the gate, by converting $N = \lfloor\frac{\texttt{allele}}{\lceil 255/N_{\rm max}\rceil}\rfloor$, where $N_{\rm max}$ is the largest number of inputs or outputs that any HMG can have. In the present implementation, $N_{\rm max} = 4$ for inputs and $N_{\rm max} = 3$ for outputs. The next $N_{\rm in}$ slots encode the identifier of the Markov variable that the HMG should read from, where identifier=$\lfloor\frac{\texttt{allele}\times 12}{255} - \frac{1}{2}\rceil$ and the symbol $\lfloor\cdot\rceil$ indicates the nearest integer. However, if a variable already has 3 or more HMGs reading from it, the connection is rerouted to the nearest available variable. Thus, alleles 10, 53, and 138 in the "From" block specify that the HMG encoded by gene 1 in Fig. S1B will read from variables 0,2, and 6. The next $N_{\rm out}$ slots specify the identifier of the Markov variable that the HMG should write to where identifier=$\lfloor\frac{\texttt{allele}\times 6}{255} + 5.5\rceil$. This ensures that the smallest identifier that a variable can write to is 6, so that an HMG can never write into a sensor. Again, not more than 3 HMGs can write into the same variable, so the connection is routed to the nearest available variable instead. According to these rules of translation, alleles 20 and 62 encode the identifiers 6 and 7 that this gate writes into.

The probabilities that specify the function of the gate are encoded in $2^{N_{\rm in}} \times 2^{N_{\rm out}}$ loci following the "From" and "To" blocks. These probabilities are determined by the relative value of alleles in each row of the transition table (see Fig. 3 as an example). So, the four alleles (127,127,127,127) in the ith row of a table with two inputs encode the probabilities $(p_{i1}, p_{i2}, p_{i3}, p_{i4}) = (1/4, 1/4, 1/4, 1/4)$, but the vector (255,255,255,255) encodes the same probabilities. Generally,

$$p_{ij} = \frac{1 + \texttt{allele}_{ij}}{\sum_{j=1}^{2^{N_{\rm out}}}(1 + \texttt{allele}_{ij})}\ . \tag{S5}$$

We add 1 to each allele in order to ensure that no probability is ever exactly zero, and in particular that the row sum is never zero. If a mutation changes $N_{\rm in}$ or $N_{\rm out}$ or both, the parser will interpret a sufficient number of subsequent loci as probabilities to fill up all the slots in the HMG table (see Fig. 3). This may happen by overlapping an adjacent gene, and because genomes are circular the parser will always succeed in assigning values.

Parsing these genes creates a network of HMGs that can be rendered either as a network of HMGs or else as a network that describes how the Markov variables interact with each other as in Fig. S1C. As an ancestral genome, we use a randomly generated genome of about 12 genes. For these genes, we randomly create the input/output connections, and fill in the probability tables (whose sizes are calculated based on the number of inputs and outputs created) using uniformly distributed random numbers $\in [0, 255]$.
