# Supplementary material for: Integrated Information Increases with Fitness in the Evolution of Animats
Source: PLoS Comput Biol. 2011 Oct 20;7(10):e1002236. doi: 10.1371/journal.pcbi.1002236 (PMC3197648; doi:10.1371/journal.pcbi.1002236)
Supplement: Text S3 — Relationship between and . (PDF) [file pcbi.1002236.s004.pdf]

Text S3. Relationship between EI and SI

In the main text we defined two measures of information integration across a partition that we called EI [Eq. (4)] and SI [Eq. (3)] which we repeat here:

$$SI(X_0 \to X_t|P) = I(X_0 : X_t) - \sum_{i=1}^{k} I(P_0^{(i)} : P_t^{(i)}) \,, \tag{S6}$$

$$EI(X_0 \to X_t|P) = \sum_{i=1}^{k} H(P_0^{(i)}|P_t^{(i)}) - H(X_0|X_t) \,. \tag{S7}$$

In this section, we derive the relationship between these two measures. We begin with arbitrary probability distributions $\Pr(X_0 = x_0)$ and $\Pr(X_t = x_t)$, and first calculate $I(X_0 : X_t)$ defined as

$$I(X_0 : X_t) = H(X_0) - H(X_0|X_t) \tag{S8}$$

where

$$H(X_0) = -\sum_{x_0} p(x_0) \log p(x_0) \tag{S9}$$

and

$$H(X_0|X_t) = -\sum_{x_0, x_t} p(x_0, x_t) \log p(x_0|x_t) \tag{S10}$$

where $p(x_0|x_t) = p(x_0, x_t)/p(x_t)$ is the conditional probability to have observed state x_0 given that we observed state x_t t time steps later. Of course, Eqs. (S8) and Eq. (2) of the main text are equivalent on account of (S9) and (S10). The relationship (S8) also holds for each of the i parts of a partition:

$$I(P_0^{(i)} : P_t^{(i)}) = H(P_0^{(i)}) - H(P_0^{(i)}|P_t^{(i)}) \,. \tag{S11}$$

If we insert (S8) and (S11) into (S6) we obtain

$$SI(X_0 \to X_t|P) = H(X_0) - H(X_0|X_t) - \sum_{i=1}^{k} \left[H(P_0^{(i)}) - H(P_0^{(i)}|P_t^{(i)}) \right] \tag{S12}$$

$$= -H(X_0|X_t) + \sum_{i=1}^{k} H(P_0^{(i)}|P_t^{(i)}) + H(X_0) - \sum_{i=1}^{k} H(P_0^{(i)}) \,. \tag{S13}$$

Together, the first two terms in Eq. (S13) are EI in Eq. (S7). The last two terms together are the negative of the (positive) integration $\mathcal{I}_{\mathcal{P}}(X_0)$ across partitions

$$\mathcal{I}_{\mathcal{P}}(X_0) = \sum_{i=1}^{k} H(P_0^{(i)}) - H(X_0) \,. \tag{S14}$$

This integration across partitions is a generalization of the quantity introduced in Eq. (13), but at step $t = 0$: Thus:

$$SI(X_0 \to X_t|P) = EI(X_0 \to X_t|P) - \mathcal{I}_{\mathcal{P}}(X_0) \,. \tag{S15}$$

For a maximum entropy distribution $\Pr^{\max}(X_0)$ (all states appear with equal probability), all possible partitions must also be uniformly distributed as there can be no correlations between them. Thus, for $\Pr^{\max}(X_0)$ (but only for this distribution)

$$\Pr^{\max}(X_0) = \prod_{i=1}^{k} \Pr^{\max}(P_0^{(i)}) \; . \tag{S16}$$

This implies that

$$H^{\max}(X_0) = \sum_{i=1}^{k} H^{\max}(P_0^{(i)}) \; . \tag{S17}$$

In that case, the integration (S14) vanishes, and EI equals SI. The same observation was made by Barrett and Seth in equations (25) and (26) of Ref. [42].
